# Bandgap engineering *via* boron and sulphur doped carbon modified anatase TiO_2_: a visible light stimulated photocatalyst for photo-fixation of N_2_ and TCH degradation†

**DOI:** 10.1039/d0na00183j

**Published:** 2020-03-31

**Authors:** Sriram Mansingh, Kundan Kumar Das, Arjun Behera, Satyabrata Subudhi, Sabiha Sultana, Kulamani Parida

**Affiliations:** Centre for Nano Science and Nano Technology, Siksha ‘O’ Anusandhan Deemed to be University Bhubaneswar 751 030 Odisha India paridakulamani@yahoo.com kulamaniparida@soa.ac.in +91-674-2581637 +91-674-2379425

## Abstract

The present research reports the synthesis of two-dimensional (2D) sheet/flake-like nanostructures of crystalline carbon modified TiO_2_ (CT), B-TiO_2_ (B-CT), and S-TiO_2_ (S-CT) using a facile one-pot synthesis method. The crystallinity and phase purity (anatase) of the prepared nano-photocatalyst were characterised using X-ray diffraction, selected area electron diffraction (SAED) and high-resolution transmission electron microscopy (HRTEM) analysis. Furthermore, the morphological details and elemental content of the sample were studied *via* scanning electron microscopy (SEM) and transmission electron microscopy (TEM), and X-ray photoelectron spectroscopy (XPS), respectively. Additionally, the optoelectronic features of all of the prepared specimens were measured *via* UV-vis diffuse reflectance spectroscopy (DRS), photoluminescence (PL), impedance and Mott–Schottky studies. After successful characterisation, their photocatalytic performance was tested towards dinitrogen photo-fixation and tetracycline hydrochloride (TCH) degradation under visible light illumination. Moreover, the effective charge separation and greater availability of the active surface area led to the robust photocatalytic activity of the fabricated B-CT compared to the CT and S-CT samples, which correlates well with the PL, impedance and surface area analysis. B-CT displays the highest photocatalytic activity, *i.e.* 32.38 μmol L^−1^ (conversion efficiency = 0.076%) of ammonia production, and 95% tetracycline hydrochloride (10 ppm) degradation. Here, we have effectively designed a novel and productive pathway towards the enhancement of the photocatalytic performance of visible photon active TiO_2_-based materials for energy and environmental sustainability.

## Abstract 2

Doped anatase TiO_2_ towards photocatalytic N_2_ fixation and TCH degradation under visible light illumination.

## Graphical abstract

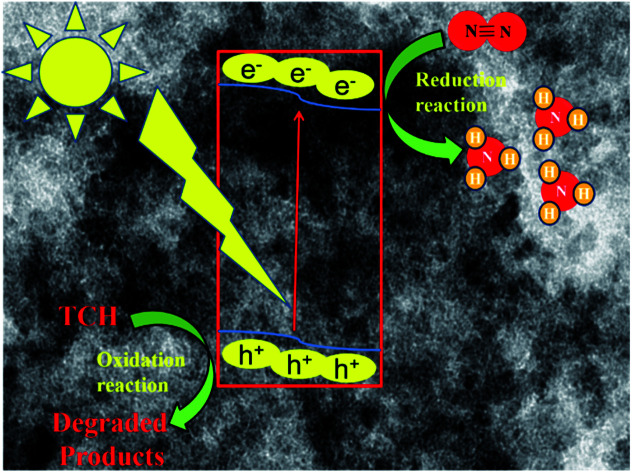

## Introduction

In the 21^st^ century, ammonia (NH_3_) is considered to be an asset towards the world economic forum in the form of feedstock, manures, fertilizer, plastics and textile industries. It is also known to be a stable carrier of hydrogen gas.^1^ Along with its economic contribution, ammonia has been considered to be an alternative energy vector because of its high density of sustainable energy like H_2_ for the upcoming generation.^2^ Furthermore, ammonia can be regarded as a replacement for non-renewable fossil fuels to meet the energy demands of the increasing population and industries. Ammonia also makes a major contribution in the biological field, as it is an important source of nitrogen in vital biomolecules such as DNA, proteins, amino acids, *etc.*^3,4^ Although NH_3_ makes a great contribution towards the well-being of society, its production by traditional methods has created environmental chaos. The commercialization of NH_3_ is possible because of the historic 19^th^-century discovery of the artificial fixation of N_2_ to NH_3_*via* the Haber–Bosch process, which propagates under extreme conditions of temperature and pressure due to the stable triple bond between the bonded N-atoms. But, the bottleneck in the reaction lies in the great use of energy and the generation of a huge amount of greenhouse gas (CO_2_) emissions.^5,6^ With the fast depletion of fossil fuels and increasing environmental problems, the production of ammonia in a sustainable way is a great challenge that faces the scientific community. In this regard, the research world has used its dextrous brain for the development of different methods, such as biological, electrochemical and catalytic, towards the reduction of dinitrogen (N_2_) to ammonia (NH_3_).^7–9^ Among these adopted techniques, photocatalytic pathway of N_2_ fixation emerges as a sustainable, green and environmentally benign process, which utilizes photon power to reduce N_2_ to NH_3_.^10–14^ In the current era, a good number of photocatalysts, such as TiO_2_, ZnO, CdS, ZnS, MoS_2_, g-C_3_N_4_, black phosphorus and BiOBr have been examined towards the photocatalytic reduction of dinitrogen.^5,15^

Of the reported photocatalysts, TiO_2_ exhibits a notable potential towards N_2_ reduction because of certain favourable features, including photostability, cost-effectiveness, non-toxicity and suitable band edge positions required for nitrogen reduction.^5^ However, the efficiency of TiO_2_ is hindered by its wide bandgap (3.2 eV) that confines its light harnessing properties to the ultraviolet region of the solar spectrum, which accounts for only 4–5% of the total light that reaches the Earth's surface.^16^ Additionally, the rapid recombination of photo excitons is another reason for its limited photocatalytic activity. Many research groups across the globe have tried their utmost to enhance the photocatalytic performance of TiO_2_ by improving its light-absorbing capacity *via* various techniques such as bandgap engineering, heterojunction formation, metal/non-metal doping, and so on.^17,18^ All these efforts have been made to enhance the photocatalytic activity by improving the light-harvesting properties, but doping with a metal or non-metal has been found to be facile and productive.^19^ Furthermore, compared to metals, non-metal addition is more promising and cost-effective, as they form intermediate band states between the two extremes, *i.e.* conduction and valence band, which extends the material light absorption range, electronegativity and, more importantly, acts as a trapping state, favouring the effective anti-recombination of exciton pairs.^19–21^ However, the problems associated with metal and noble metal doping are that the materials undergo a bandgap shift that leads to faster recombination, photo-corrosion reducing stability and lastly the synthesis procedure becomes expensive. Also, metal ion doping leads to thermal instability and the formation of sub-bands that act as exciton recombination sites and hence minimise the catalytic performance of the photocatalysts. However, the introduction of anions into the lattice results in an upward movement of the valence band, narrowing the bandgap energy and creating anion vacancies with a stable cubic or tetrahedral phase of the metal oxide system under atmospheric conditions.^22^ So, non-metal introduction into the TiO_2_ lattice is the best way to drive UV-TiO_2_ to the visible region and also suppress the charge recombination mechanism.^23,24^ Additionally, carbon doping forms hybrid levels and sulphur addition results in the formation of localized states that ultimately reduce the optical bandgap and increase the photon absorption range,^25^ whereas highly electronegative non-metal (*e.g.* fluorine) doping generates sub-bands below the valence band and widens the optical energy gap.^19^ In terms of anion-doped TiO_2_ photocatalysts, our groups have made a significant contribution.^26–29^

Taking all the above knowledge from the reported literature, herein we have developed carbon modified TiO_2_, S-doped carbon modified TiO_2_ and B-doped carbon modified TiO_2_*via* a simple solvothermal method. Furthermore, to the best of our knowledge, this is the first ever report on the photoreduction of dinitrogen to ammonia using a one pot synthesis of visible active TiO_2_ nanosheets. Additionally, their catalytic activities were also tested towards tetracycline hydrochloride photodegradation and the fragmentation pathway was verified *via* liquid chromatography mass spectrometry (LC-MS) analysis. The phase purity and morphological modifications of the as-synthesized photocatalysts were analysed by X-ray diffraction (XRD) and transmission electron microscopy (TEM) studies. The effective charge carrier separation and large exposed surface sites were well characterised *via* photoluminescence (PL), electrochemical impedance spectroscopy (EIS) and Brunauer–Emmett–Teller (BET) analysis. The existence of sulphur and boron in the respective doped carbon modified TiO_2_ was confirmed from the XPS and elemental mapping. This finding will project some new ideas towards the photocatalytic application of modified TiO_2_.

## Characterization

The detailed crystallographic features of the synthesised samples were analysed with the help of a Rigaku-Ultima IV XRD instrument equipped with a Cu Kα radiation source (*λ* = 0.154 nm) in the 2*θ* scan range of 10°–80°. A JASCO V-750 UV-VIS spectrophotometer was used to estimate the optical behaviour in the range of 200–800 nm. Furthermore, the PL properties were characterised using a JASCO spectrofluorometer FP-8300. The peripheral morphologies of S-CT, B-CT and CT were investigated by SEM using a ZEISS SUPRA-55 microscope. Additionally, the internal topological details of the photocatalyst were studied using TEM (200 kV power) using a JEOL JEM 2100 microscope. XPS measurements were undertaken using a VG microtechmultilab ESCA 3000 spectrometer fitted with a non-monochromatic Mg-Kα X-ray radiation lamp to determine the chemical environment of the material. The BET surface area and Barrett–Joyner–Halenda (BJH) pore size distribution studies of the samples were carried out using NOVA2200e Quantachrome Apparatus. A multi-channel framed IVIUM-n-STAT electrochemical analyser was used for electrochemical measurements of the as-synthesized materials. To trace the intermediate products of TCH degradation, LS-MS analysis was performed using a TSQ Quantum Access MAX triple quadrupole mass spectrometer, Thermo Fisher Scientific.

### Electrochemical characterization

The electrochemical behaviour of the as-prepared photocatalysts was characterised using a conventional three electrode-based quartz cell containing 0.2 M Na_2_SO_4_ as the electrolyte, Pt as the counter electrode, saturated Ag/AgCl as the reference electrode and catalyst-coated fluorine-doped tin oxide (FTO) glass as the working electrode. A drop casting method was generally used to make the working electrode, where the FTO was first rinsed with deionised water (DW) and ethanol by sonication for nearly 30 min to clean the surface and then dried at 100 °C in a hot air oven. Next, the above polished FTO plates (conducting surface) were coated with a pasty mass containing a mixture of the sample, ethanol, binder (Nafion) and DW. The slurry was prepared by mixing the photocatalyst (1 mg) + ethanol (1.4 mL) + Nafion (40 mL) + DW (1.6 mL) and then subjecting this suspension to sonication for 30–45 min to ensure proper mixing. Then, this slurry was applied to the FTO, which was dried in a vacuum oven.

## Experimental section

### Chemicals used

1-Octadecene (90%) and TiCl_4_ were purchased from Sigma Aldrich. Sulphur (S) and boric acid (H_3_BO_3_) were purchased from Merck and used as received.

### Synthesis procedure

**Scheme 1:**
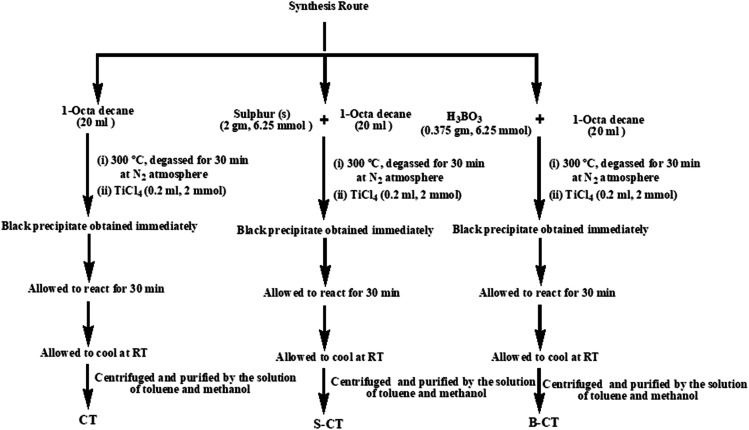
Schematic representation of the synthesis of S-CT, B-CT and CT.

The synthesis was carried out as follows. 20 mL of 1-octadecene (90%, Aldrich) was added to 2 g (6.25 mmol) of sulfur and was stirred for 30 min. The resulting suspension was then heated up to 300 °C for 30 min before adding 0.2 mL (1 mmol) of TiCl_4_ into the rapidly stirring sulphur solution and the mixture immediately turned black. The total experiment was conducted under a N_2_ atmosphere, where the solution was kept under the same conditions for 30 min and then allowed to cool to room temperature. Then, the resulting solution was centrifuged and purified by washing it with a solution of toluene and methanol repeatedly seven times. The black precipitate obtained was dried under vacuum at 80 °C to obtain S-doped carbon modified titania (S-CT). B-Doped carbon-modified titania (B-CT) was prepared using the same procedure instead using 0.375 g (6.25 mmol) of boric acid (H_3_BO_3_) in place of sulphur. Carbon-modified TiO_2_ (CT) was prepared using the same procedure without sulphur or boric acid. Scheme 1 shows the adopted synthesis procedure.

### Photocatalytic nitrogen reduction

The catalytic supremacy of the prepared photocatalysts was tested for the photoreduction of N_2_ to NH_3_ under a visible light-emitting 250 W medium pressure Hg lamp (intensity = 1330 mW) with a cut-off filter (≥420 nm) for 3 h. In a typical experiment, 15 mg of the photocatalyst was added to 50 mL of DW in a quartz batch reactor fitted with a chiller to maintain an inner temperature of around 25 °C. Furthermore, the reaction mixture was slowly bubbled with N_2_ gas for nearly 1 h to saturate the solution with N_2_. Then, it was stirred in the dark for 1/2 h to develop an adsorption–desorption equilibrium. After this, the light source was switched on and at a calculated time gap, 5 mL of the treated reaction solution was withdrawn and at once centrifuged to remove the catalyst. Moreover, another quartz container with dilute H_2_SO_4_ was used to trap the NH_3_ carried by the gas-phase effluent and later analysed. While calculating the total amount of ammonia formed, NH_3_ contained in both the reactor and trapping vessel were taken into consideration. An indophenol blue method was followed to quantify the amount of NH_3_ produced. The optical absorbance of the as-developed coloured solutions was characterised using UV-vis spectroscopy. Again, quantification of the ammonia and hydrazine amount was analysed with the previously made standard calibration curves of each complex. A blank analysis was carried out in the absence of catalyst, *i.e.* only of the complexing reagents and N_2_ saturated DW.

## Results and discussion

### X-ray diffraction analysis (XRD)

**Fig. 1:**
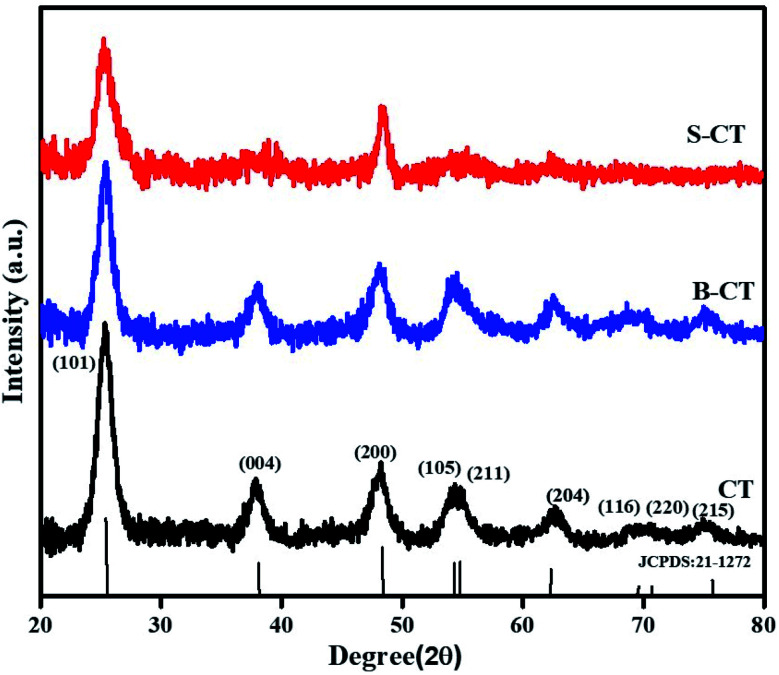
XRD patterns of CT, B-CT and S-CT.

The X-ray diffraction pattern in Fig. 1 represents a pure anatase crystal phase for all of the prepared samples, *i.e.* carbon modified TiO_2_, B-CT and S-CT, without any additional TiO_2_ phase (rutile or brookite). The observed Bragg's diffractogram at 2*θ* = 25.55°, 37.86°, 48.04°, 54.29°, 67.72°, 69.49°, 70.71° and 75.38° was indexed to the (101), (004), (200), (105), (204), (116), (220) and (215) crystal planes, respectively, of anatase TiO_2_ which corresponds to JCPDS 21-1272.^30,31^ Furthermore, it was clearly visible that upon the addition of dopant (S and B atoms) to CT, the sharpness of the XRD peaks was reduced notably and there was also a shift in the 2*θ* value, which implies a good lattice interaction and increase in the crystallite size or in other words, the added species enters the lattice framework. From the plotted data, it was obvious that the introduction of S and B atoms into the TiO_2_ lattice resulted in a suppression of crystal nucleation, as observed *via* the peak broadening and decrease in intensity, *i.e.* a distortion of the crystal lattice.^32^ Again, the peak broadening also indicates a reduction in particle size, which was well supported by TEM analysis. As discussed above, doping of S and B into TiO_2_ causes a minute shift in peak position, which can be attributed to the difference in electronegativity and radius of the substituted atom and substituting atoms.^33^ Interaction between the added dopant results in intrinsic defects or anion vacancies. Furthermore, a detailed crystallographic parameters are shown in Table 1, which provides information regarding the mean crystallite size (calculated using Scherrer and Williamson–Hall (W–H) formulae), micro-strain and dislocation density.^31^

**Table 1:** Crystallographic parameters

Sample name	The average crystallite size (*D*) (nm)		Lattice strain (*ε*) × 10^−3^	Dislocation density (*δ*)
	Scherrer's	W–H		
CT	0.96	1.24	0.00035	0.6536
B-CT	1.02	1.31	0.00033	0.5848
S-CT	0.88	1.38	0.00039	0.5263

### Chemical composition (XPS) analysis

**Fig. 2:**
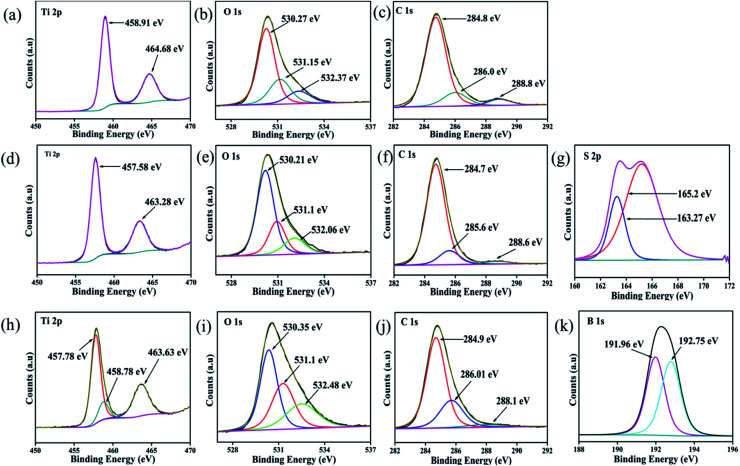
XPS spectra of (a, b and c) Ti 2p, O 1s and C 1s of CT, (d, e, f and g) Ti 2p, O 1s, C 1s and S 2p of S-CT (h, i, j and k) Ti 2p, O 1s, C 1s and B 1s of B-CT respectively.

To examine the elemental content, respective oxidation state and their corresponding electronic environment of all the developed photocatalysts, XPS measurements were carried out. The core-level XPS spectra of Ti 2p, S 2p, B 1s, O 1s and C 1s are shown in Fig. 2. The full survey scans of each sample, *viz.* CT and doped CT, along with the XPS spectra of unmodified anatase TiO_2_, are shown in Fig. S1 (ESI†). Fig. 2a depicts the deconvoluted XPS spectra of Ti 2p, where the peaks positioned at 464.68 and 458.91 eV represent the Ti 2p_1/2_ and Ti 2p_3/2_ spin states, further suggesting the presence of Ti in a +4 oxidation state.^16^ In the case of S-doped CT (Fig. 2d), the Ti 2p_1/2_ and Ti 2p_3/2_ spin states of Ti can be observed at 463.28 and 457.58 eV. However, a negative shift in the Ti peaks can be observed in the case of S-doped CT, which could be ascribed to the partial replacement of oxygen atoms from TiO_2_ by S ions of lower electronegativity. The replacement of oxygen leads to an increase in the charge of Ti atoms and subsequently decreases the binding energy of Ti 2p.^34^ In B-doped CT (Fig. 2h), Ti 2p_1/2_ and Ti 2p_3/2_ are located at 463.63 and 457.78 eV. However, in the case of boron, a peak for titanium can be observed at a higher binding energy of 458.78 eV, which can be ascribed to the formation of a Ti–B species. Fig. 2b, e and i displays the O 1s deconvoluted XPS spectra of CT, sulphur and boron-doped CT. In carbon-modified TiO_2_, two peaks for O 1s can be observed at binding energies of 530.27 and 532.37 eV, which can be ascribed to the lattice oxygen (Ti–O–Ti), Ti–OH groups and surface H_2_O (absorbed oxygen), respectively. Moreover, the peak observed at 531.15 eV can be correlated with an oxygen vacancy, which plays a crucial role in the catalytic process as it acts as an electron trapping centre and also a substrate anchoring site.^16,35^ Similar types of bands were observed for sulphur and boron-doped carbon-modified TiO_2_, with a slight shift in the peak position. For S-doped CT, the peaks located at binding energies of 530.21, 531.12 and 532.06 eV can be assigned to Ti–O, S–Ti–O and S–O bonds, respectively, indicating that some of the O atoms in TiO_2_ have been replaced by S atoms.^36^ For B-doped CT, the peaks positioned at 530.35 eV correspond to the Ti–O and Ti–O–B bonds and the peak centred at 532.48 eV arises due to the B–O–B and surface adsorbed water, while the peak centred at 531.1 eV indicates the displacement of oxygen in the TiO_2_ lattice.^37,38^ Similarly, Fig. 2c, f and j displays the C 1s spectra of CT and doped CT. The carbon spectra of TiO_2_ can be deconvoluted into three peaks which are located at 284.8, 286 and 288.8 eV. The peak at 284.8 eV can be ascribed to the C–C bond, which covers a maximum area of a C 1s peak. The bands at 286 and 288 eV can be assigned to C–O/C–OH and C–OH bonds, respectively. Fig. 2f displays three deconvoluted XPS peaks of S-doped CT positioned at 284.7, 285.6 and 288.6 eV, which could have originated from C–C, C–O and C=O, respectively. Similar trends were also observed in the case of boron-doped CT. However, no carbon phase was observed in the XRD spectrum, but the appearance of carbon peaks in the XPS spectra supports the entry of carbon into TiO_2_ as a dopant. The S 2p core level deconvoluted spectra, as displayed in Fig. 2g, represents two XPS peaks at 163.27 and 165.19 eV and can be ascribed to the S 2p_3/2_ and S 2p_1/2_ spinal states, which further confirms the presence of sulphur in the S^2−^ and S^4+^ states in the TiO_2_ lattice.^39,40^ Additionally, the band at 163.27 eV represents the development of the Ti–S bond. It can be suggested that sulphur replaced the oxygen atoms in the TiO_2_ framework.^34^ However, on deconvolution, we did not find any band above 165.5 eV, which confirms that sulphur was not been oxidized to sulphate and sulphite groups.^34^ The observed XPS peaks between 190 and 194 eV are because of the B 1s electronic level, which can again be deconvoluted into two peaks at binding energies of 191.96 and 192.75 eV, as shown in Fig. 2k. The first one is related to the B atom going into the TiO_2_ lattice and replaces the oxygen site to form O–Ti–B bond.^38^ However, the higher one is attributed to the Ti–O–B bond.^41^ Again, the elemental composition, as shown in the XPS of each sample, is well supported by the EDX and colour mapping images discussed in the respective sections.

### UV-vis DRS

**Fig. 3:**
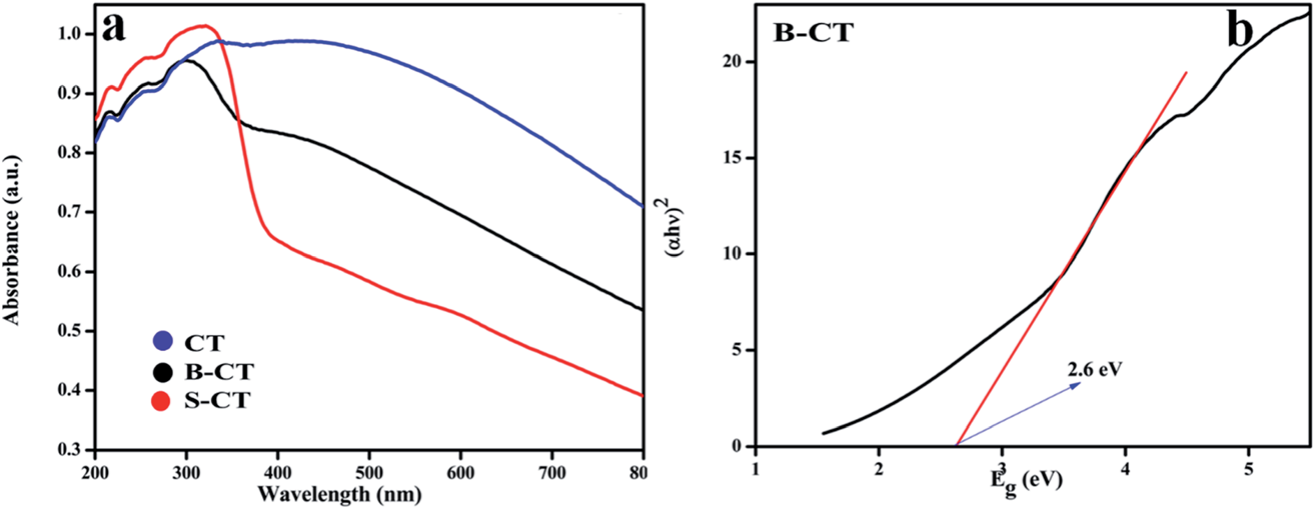
(a) UV-Vis absorption spectra of CT, S-CT and B-CT and (b) the band gap energy of B-CT.

The light responsive properties of the catalyst play a crucial role in the process of photocatalytic reactions. To verify the light-sensitive characteristics of all of the synthesised photocatalysts, UV-vis DRS analysis was performed and the obtained absorbance values are shown in Fig. 3a. From the plotted optical absorbance graph, it can be clearly seen that CT (carbon-modified TiO_2_) has a wide light harnessing ability that covers the whole visible and part of the infrared region of the solar spectrum, which is due to the carbon presence as carbon shows black body properties. However, a new phenomenon is visualised, *i.e.* when CT is doped with the B or S atom, the absorbance experiences a lower wavelength region (hypochromic shift) drift, as shown in Fig. 3a, which is due to the doping effect. Moreover, the bandgap energy of the B-CT photocatalyst was calculated using the Kubelka–Munk eqn (1):

1 *αhν* = *A*(*hν* − *E*_g_)^*n*^

where *h*, *α*, *E*_g_, *ν*, and *A* are Planck's constant, optical absorption coefficient, bandgap energy, frequency of light and the proportionality constant, respectively. For the above equation, *n* indicates the type of optical transition of the photocatalyst, *i.e.*, *n* = 1/2 for a direct transition and *n* = 2 for an indirect transition.^42^ The band-gap energy of the B-TiO_2_ photocatalyst is 2.6 eV (Fig. 3b) and those of CT and S-CT are depicted in Fig. S2.† Furthermore, the calculated Urbach energies for CT, B-CT and S-CT are 1.31, 1.95 and 1.81 eV and the bandgap energy of CT and S-CT, respectively, are shown in Fig. S3 (ESI†). The higher value for B-CT implies that there are more disorders/defects in the TiO_2_ lattice. All the calculations were made following our previously reported work.^43^

### Photoluminescence spectroscopy (PL) analysis

**Fig. 4:**
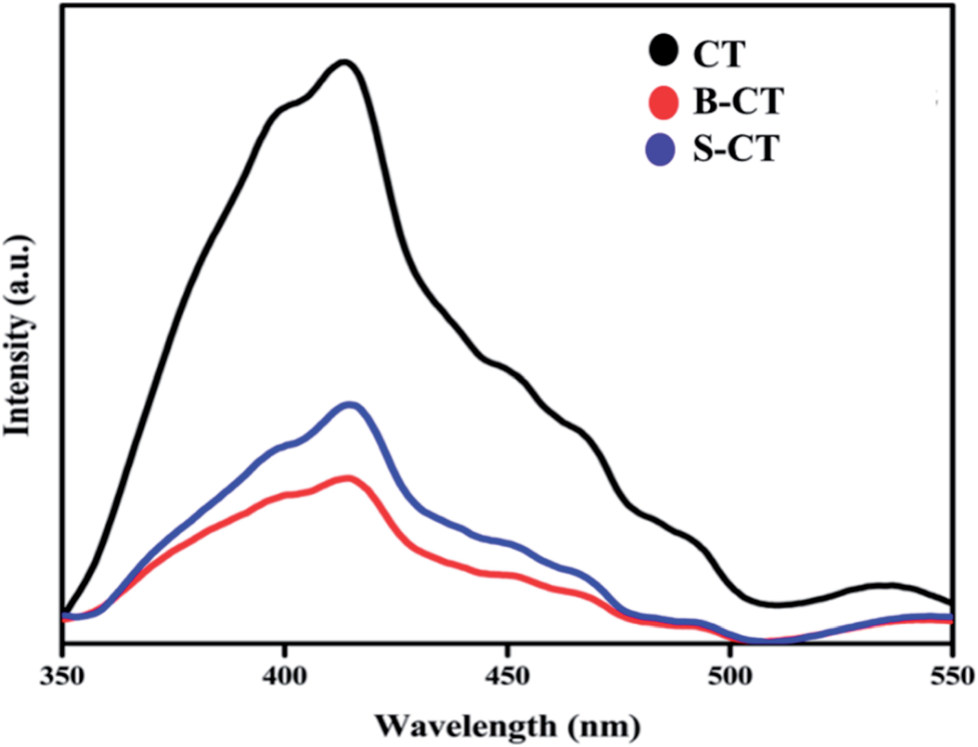
Photoluminescence spectra of CT, S-CT and B-CT.

Another important optical characterization technique is PL spectroscopy, which gives vital information regarding the photogenerated e^−^/h^+^ pair recombination and transfer behaviour.^33^Fig. 4 depicts the PL analysis results of carbon-modified TiO_2_ and its doped counterparts. It was observed that the PL peak of doped TiO_2_ is of low intensity compared to that of CT (B-CT < S-CT < CT). The different excitonic transition that occurs in the photocatalyst is due to the oxygen vacancies and surface defects of the TiO_2_ photocatalyst. The PL spectra centred at 399 nm is related to the light responsive electronic transition between the valence band and conduction band of TiO_2_ while the other peaks centred at 413, 451, 469, 490 and 532 nm can be allocated to the oxygen vacancies of self-trapped electrons, recombination of photogenerated electrons and holes and the intrinsic state of TiO_2_.^44^ In particular, the peak that arises at 413 nm is due to the indirectly allowed transition of excitons at TiO_6_ octahedral sites, while the peak at 451 nm is due to the charge separation between Ti^3+^ and O_2_^−^ in (TiO_6_)^8−^.^45^ Furthermore, the peaks at around 469, 490 and 532 nm originate from the surface defects and oxygen vacancies of the TiO_2_ photocatalyst, respectively.^46^ These defect states act as trapping sites and hence reduce the exciton recombination process and prolong the lifetime of charge carriers. Again, effective separation means a greater number of photogenerated electrons and holes, and hence more catalytic activity. The above claim of productive charge separation in B-CT was well justified from the EIS study and photocatalytic activity.

### Electrochemical impedance spectroscopy (EIS) analysis

**Fig. 5:**
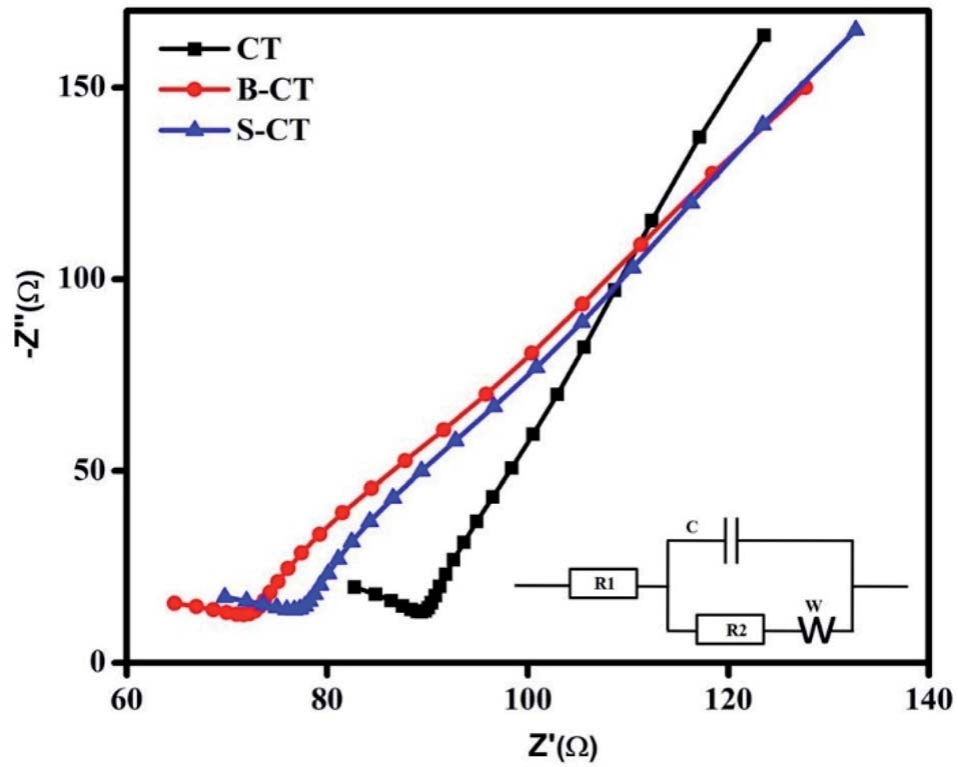
Nyquist plots of CT, S-CT and B-CT.

So far, we have discussed the exciton separation efficiency through observing the radiative emission of samples, *i.e.* PL analysis. Now to add more to the above phenomenon, such as charge carrier separation and the effective transfer of photogenerated e^−^–h^+^ pairs to carry out the photocatalytic reaction, EIS measurements were performed under zero basing conditions in the dark of the as-synthesised CT and doped CT. The plotted Nyquist graph in Fig. 5 is divided into two parts (i) a higher frequency region containing the semi-circle gives an idea about the pace of charge separation and transfer phenomenon and (ii) the second part in a lower zone (loop) gives information about the electron transport kinetics and ion diffusion rate.^47^ Generally, the smaller the diameter of the arc, the better the charge separation and transfer effect. Similarly, the straighter the loop, the faster the electron migration and ion diffusion kinetics.^30,48,49^ In the present investigation, doped CT (B-CT) shows an effective separation and better transport of the charge carriers (small arc diameter and straight loop) compared to S-CT followed by CT, as observed from the Nyquist graph. Additionally, the inset in Fig. 5 represents a suitable circuit diagram that fits the plotted Nyquist graph where *R*_1_ and *R*_2_ stand for the electrolyte and electrolyte/electrode resistance and *C* and *W* represent the capacitance and Warburg impedance value. Moreover, the above conclusion is well supported by the PL and catalytic activity studies.

### Mott–Schottky (MS) analysis

**Fig. 6:**
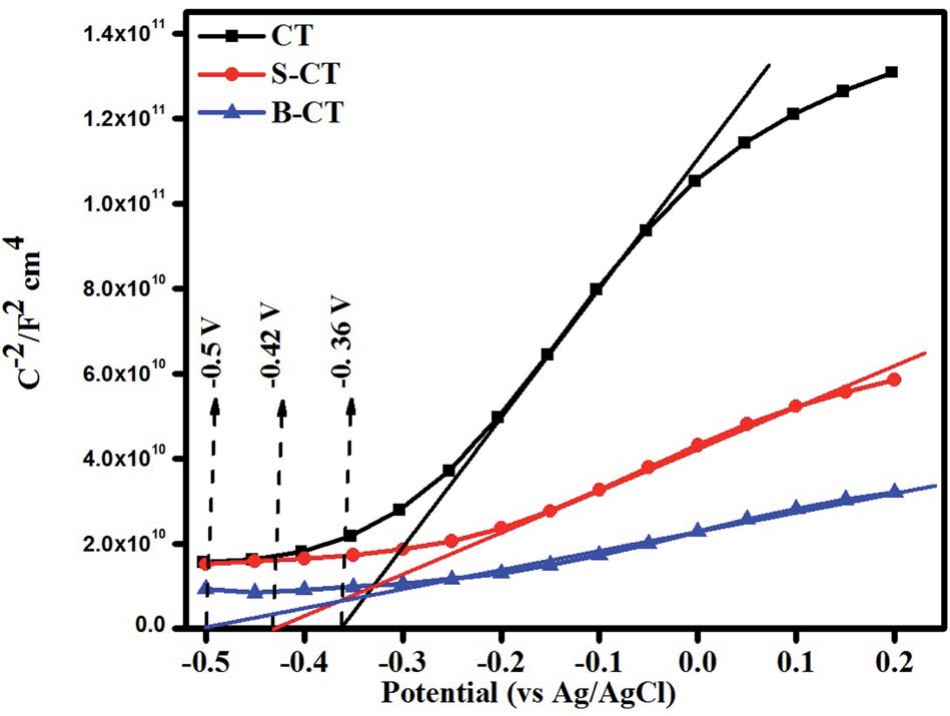
Mott–Schottky plot of CT, S-CT and B-CT.

Mott–Schottky analysis was carried out to determine the nature of the semiconductor (n or p-type) and to compute the band edge potential which will be used in sketching out the reaction mechanism. The flat band potential was calculated following the Mott–Schottky formula referring to our published article.^50^Fig. 6 depicts the MS plot of the synthesised photocatalyst scanned in the dark at an applied frequency of 500 Hz. The positive slope drawn on the potential axis for all of the samples implies an n-type semiconducting feature, *i.e.* electrons as the majority charge carriers and the calculated flat band potentials are found to be CT = −0.36 V, S-CT = −0.42 V and B-CT = −0.5 V *vs.* Ag/AgCl.^51^ The intercept on the *X*-axis gives the flat band potential (*E*_fb_) value and in the case of n-type semiconductors, the bottom of the conduction band (CB) lies just 0–0.1 V above the *E*_fb_. So, we can say that the conduction band potential of B-CT is −0.41 eV and the VB is 2.19 eV *vs.* the NHE scale at pH 6.8 as the *E*_g_ of B-CT is 2.6 eV.^43^ Furthermore, it was observed that in doped systems the *E*_fb_ undergoes a negative shift/smaller slope, suggesting a higher donor density, which can be attributed to the presence of oxygen vacancies/defects (proved *via* XPS and the Urbach energy) and this will lead to effective separation and transport of charge carriers about the electrode–electrolyte interface.^52^ Additionally, as the B-doped carbonised TiO_2_ has a higher negative *E*_fb_ value, it will show higher charge conductivity and mobility that ultimately boost the catalytic activity.

### SEM and HRTEM analysis

**Fig. 7:**
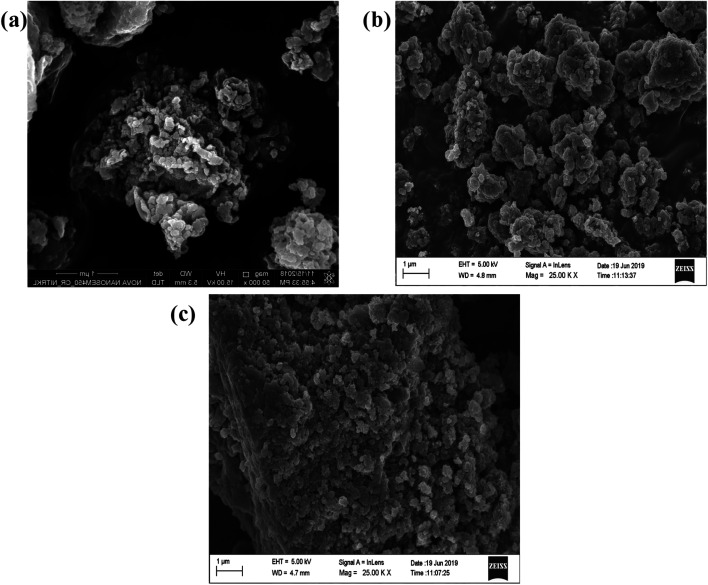
FESEM images of (a) CT, (b) B-CT and (c) S-CT.

**Fig. 8:**
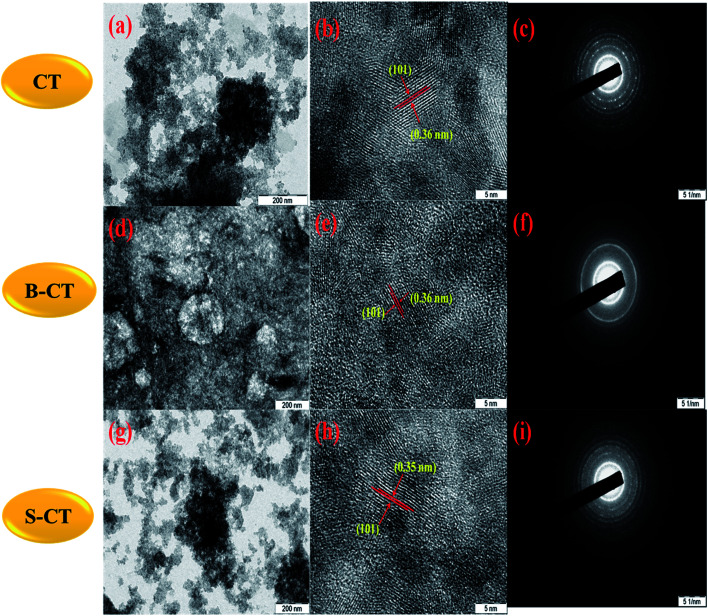
TEM, HRTEM and SAED images of ((a), (b) and (c)) CT, ((d), (e) and (f)) B-CT and ((g), (h) and (i)) S-CT.

In order, to ascertain the shape, size, elemental content and crystallinity of the as-prepared material, SEM, TEM, HRTEM, SAED and EDX analyses were performed. The SEM image shown in Fig. 7a presents a sheet-like morphology for CT. However, more agglomeration and a fragmented sheet-like morphology were observed in the case of S- and B-doped CT (Fig. 7b and c). The agglomeration of sheets may be due to the existence of van der Waals forces of attraction, whereas the TEM of CT depicts the sheets more clearly, in the case of doped carbonised TiO_2_ (S- and B-doped), the sheets were more or less of the fragmented type with a number of white spots that indicate poor formation on the sheet horizon. Furthermore, the lattice *d*-spacing as calculated for all of the prepared samples (CT, B-CT and S-CT) were found to be *d* = 0.36 nm and indexed to the (101) crystal plane of anatase TiO_2_. Fig. 8c, f and i displays the SAED images of doped and un-doped TiO_2_. The definite circular ring along with dots implies the polycrystalline character of the prepared samples and these data show good correlation with the performed XRD analysis. The elemental content, such as Ti, O, S, B and C, was well proved *via* EDX analysis, which was further confirmed through colour elemental mapping that shows the elemental distribution in the different as-prepared samples and detailed images are shown in the ESI (S3 and S4†).

### N_2_ adsorption–desorption analysis

**Fig. 9:**
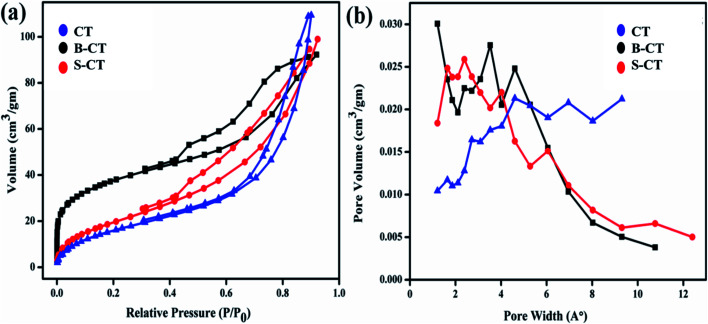
(a) Nitrogen sorption isotherms and (b) pore size distribution curves of CT, S-CT and B-CT.

As we know, surface area plays a vital role in magnifying the catalytic potential of photocatalytic materials, so BET and BJH measurements, *i.e.* N_2_ the adsorption–desorption isotherms and pore size distributions of the as-prepared samples were carried out and are presented in Fig. 9. It can be clearly visualised from the plotted BET results that all of the studied photocatalysts depict a type-IV isotherm with a H_2_ hysteresis loop, which implies a mesoporous framework of aggregated small TiO_2_ crystallites (well supported by the TEM images).^53^ Furthermore, in the BJH graph, peaks were observed at a pore diameter of 8.08 nm (CT), 11.12 nm (B-CT) and 4.35 nm (S-CT), indicating the narrow distribution of the mesopore dimensions. Moreover, all of the collected data regarding pore volume, surface area and pore diameter distribution of the prepared samples are tabulated in Table 2. From the above-obtained results, it was confirmed that B-doped CT has a higher surface area with a larger pore diameter and volume compared to CT. This is because doping of non-metals/anions into the TiO_2_ lattice prevents the aggregation of smaller TiO_2_ crystallites, resulting in a bigger pore and higher surface area. But, in the case of CT, the small TiO_2_ aggregates to form sheets that are small in size.^32^ The high surface area of doped TiO_2_ is well reflected in its high catalytic performance. This further clarifies that high light absorption is not the only criterion that determines the photocatalytic superiority of the material, the surface area also plays a major role.

**Table 2:** Representation of the obtained surface area, mean pore diameter and pore volume

Sample	Surface area (m^2^ g^−1^)	Mean pore diameter (nm)	Pore volume (cm^3^ g^−1^)
B-CT	127.28	11.12	0.16
S-CT	60.87	5.62	0.20
CT	75.74	8.08	0.17

### Photocatalytic reduction of N_2_ to NH_3_

**Fig. 10:**
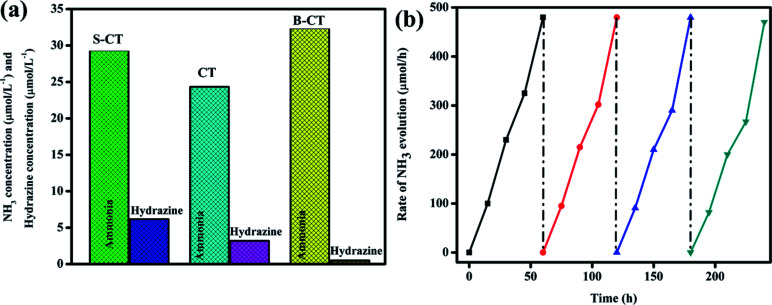
(a) Photo fixation of nitrogen to ammonia and to hydrazine over CT, S-CT and B-CT and (b) reusability graph of B-CT.

The catalytic ability of the designed photocatalysts was examined towards the reduction of dinitrogen to ammonia under visible light illumination. In these experiments, 0.15 g of the sample was added to 50 mL of deionised water (DW) saturated with N_2_ gas and in the absence of a scavenging reagent. A series of other experiments were performed, *i.e.* in the absence of a catalyst or light, no ammonia formation was observed, which confirms that ammonia generation only occurs *via* the photocatalytic pathway. It can be seen from Fig. 10a that CT displays a low catalytic activity with 24.33 μmol L^−1^ whereas B-CT and S-CT produce NH_4_^+^ with 32.38 and 29.33 μmol L^−1^, respectively. The above reduction experiment was carried out in a methanol solution (10 vol%) and in a low pH range; detailed effects of the used reagents and conditions are given below. This enhancement in the yield rate due to CH_3_OH use can be attributed to it (i) acting as an electron donor and (ii) producing CO_2_, as both these factors favour N_2_ fixation. Additionally, it was observed that N_2_ reduction is more feasible under a low pH as the activation energy barrier is reduced under acidic conditions and a greater number of protons will be in the medium to accelerate the conversion process. Hence, the photoreduction reaction was performed for the as-prepared samples in an acidic medium by adjusting the pH with dilute HCl. Out of them all, B-CT shows the best result due to the effective separation of charge carriers *via* defect sites and a porous framework that act as anchoring points for N_2_ and its further reduction to ammonia. Hence, under a low pH or in a high proton concentration, catalysts are more active towards N_2_ photoreduction. Again, the apparent conversion efficiency (solar to chemical) of B-CT was found to be 0.076%.^54^

Furthermore, to justify the importance of N_2_ gas, DW and photoelectrons, more reactions were set up such as (i) no ammonia was obtained when the reaction was carried out in argon saturated DW, which suggests that the photoreduction of the supplied N_2_ gas takes place specifically, (ii) again, when the aprotic solvent DMF was used still no ammonia formation was seen, which implies the protons of water aid in the photo fixation process, and finally, (iii) with the addition of AgNO_3_ (an electron scavenger) a very low or negligible amount of ammonia was produced, suggesting the importance of electrons in the conversion of N_2_ to NH_3_. Furthermore, longevity or stability is a vital parameter in quantifying the efficiency of the catalyst, and hence the best photocatalyst (B-CT) was again subjected to a reusability test and it can be observed in Fig. 10b that the material maintains its activity for up to three consecutive cycles and then starts to reduce in the fourth cycle.

### Photodegradation of TCH

**Fig. 11:**
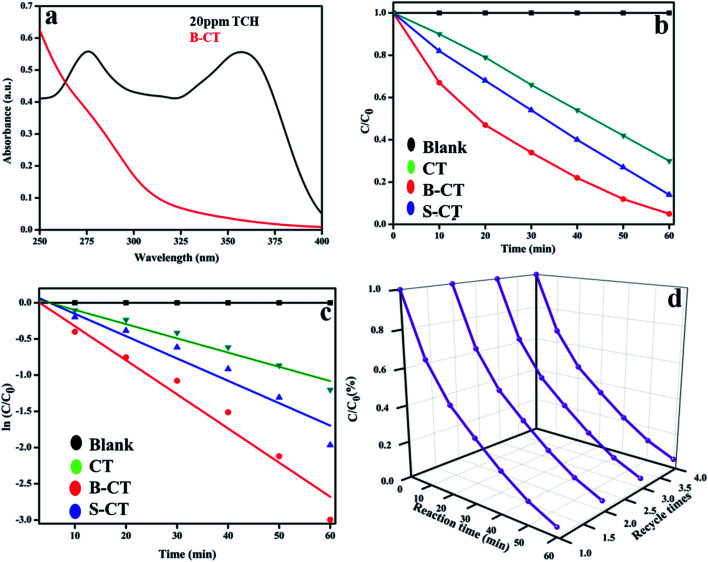
(a) Optical absorption spectra of neat TCH and B-CT treated TCH, (b) *C*/*C*_0_ plot of CT and doped CT, (c) kinetics plot of CT and doped (B and S) CT and (d) a graph showing the photostability of B-CT.

Furthermore, to aid the photocatalytic potential, as shown in Fig. 6, measurements were again carried out for the decomposition of TCH (20 mL (10 ppm) TCH + 20 mg catalyst) under visible photon irradiation (250 W medium pressure Hg-lamp) in the same quartz batch reactor setup. Fig. 11a displays the optical absorbance graph of neat TCH and B-CT photocatalysed TCH. Furthermore, it was found from the *C*/*C*_0_ as highlighted in Fig. 11b that B-CT shows the best degradation results, *i.e.* 95%, with S-doped at 86% and CT at 70% within a time gap of 1 h. The plotted absorbance graph depicts two peaks, one at 275 nm (acylamino and a hydroxyl group) and the other at about 357 nm (hydroxyl-containing rings). Again, the degradation kinetics, *i.e.* the ln *C*/*C*_0_*vs.* time plot (Fig. 11c) follows pseudo first-order kinetics with a high rate contact value for B-CT (see Table 3). Furthermore, to examine the photo durability, which is an important physical parameter in distinguishing the material photocatalytic ability from other properties, a catalyst reusability experiment was performed following our previously reported paper and the obtained data are plotted in Fig. 11d. The results suggest that the catalyst is quite stable up to four consecutive cycles with a very small decrease in the activity, which can be attributed to mass loss and active surface deterioration during the cleaning process for further use. Additionally, to showcase the robust photocatalytic performance of our prepared photocatalyst with those of other reported doped TiO_2_ photocatalytic systems, a comparison table is in included in the ESI (T1†).

**Table 3:** TCH degradation details

Photocatalyst	*R* ^2^	Rate constant ‘*k*’ (min^−1^)	Half-life period *t*_1/2_ (min)	TCH degradation (%)
B-CT	0.95	47 × 10^−3^	14.74	95
S-CT	0.93	30 × 10^−3^	23.1	86
CT	0.95	19 × 10^−3^	36.47	70

### Scavenger test

**Fig. 12:**
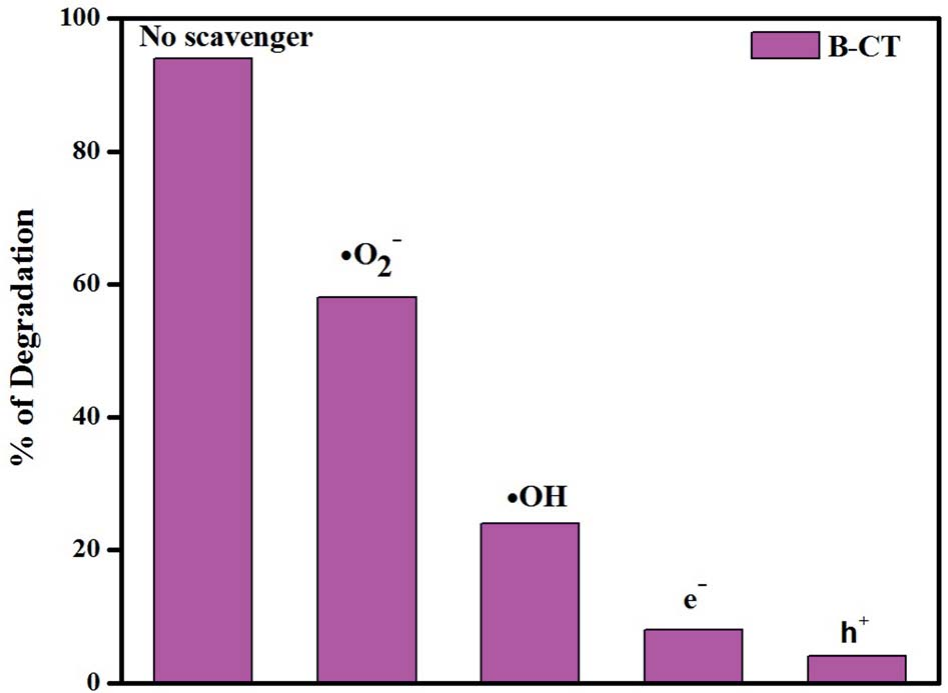
Histogram showing the scavenger test results.

In order to elaborate the reaction pathway (a mechanism), *i.e.* the active species (h^+^, e^−^, OH˙, O_2_˙^−^) responsible for the photocatalytic reaction, and hence draw a proper scheme of the mechanism, scavenger experiments were carried out and the details are described as follows. In the performed test, tracing reagents were taken for specific active species, such as benzoquinone (˙O_2_^−^), isopropyl alcohol (˙OH), EDTA (h^+^) and AgNO_3_ (e^−^). In the case of antibiotic degradation, it was found that with the addition of isopropanol and benzoquinone, the degradation percentage significantly reduces. This observation clearly suggests that hydroxyl (˙OH) and super-oxide (˙O_2_^−^) radicals play a key role in the photodegradation process. Fig. 12 clearly supports the above claim, wherein the presence of a superoxide and the hydroxyl maximum degradation is shown.

### The LC-MS analysis of the TCH photodegradation over B-CT

**Scheme 2:**
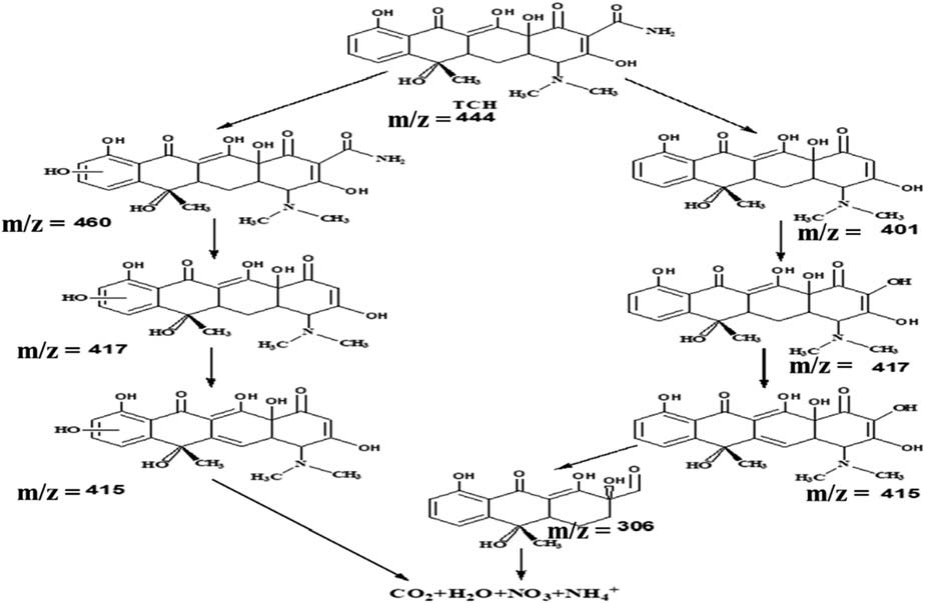
The degradation pathway *via* different intermediates.

To gain more insight about the degradation pathway of TCH over a B-CT surface under light irradiation, LC-MS characterization was carried out. Based on the obtained MS spectrum as shown in ESI (S5†), a fragmentation pathway of TCH photo-degradation *via* different intermediate species identified by their respective *m*/*z* ratio is presented in Scheme 2.

### Mechanistic approach

**Scheme 3:**
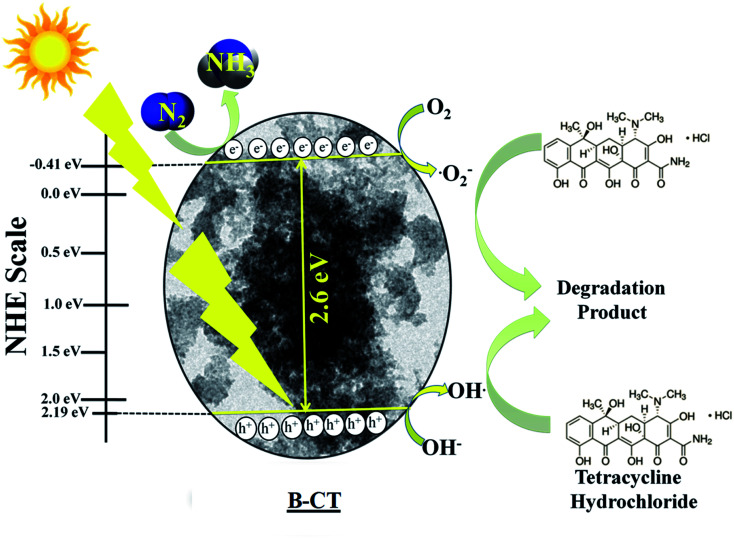
The mechanism of N_2_ photo-fixation and TCH degradation over B-CT.

To explain the high photocatalytic activity of B-doped CT towards N_2_ reduction and TCH degradation under visible light illumination, we have designed a plausible mechanistic pathway, as depicted in Scheme 3. From the Mott–Schottky analysis, the CB position of B-CT was estimated to be −0.41 eV and VB is about 2.19 eV on the NHE scale (at pH 6.8). Upon light irradiation, the electrons in the VB of B-CT get photoexcited and accelerate to the CB leaving behind holes. The gathered photoelectrons on CB come into action and start reducing N_2_ to NH_3_ along with protons generated from the acidic solvent. Also, these highly reducing electrons interact with molecular oxygen and produce a superoxide radical as the CB band position is highly negative (−0.41 eV) compared to the standard redox potential (O_2_/˙O_2_^−^ = −0.33 eV *vs.* NHE).^43,55,56^ This favours the production of a high amount of superoxide radicals which then react with TCH and initiate the degradation process. Furthermore, the holes in the VB (2.19 eV) are capable of generating hydroxyl radicals (OH/OH˙ = 1.99 eV *vs.* NHE) due to their high reduction potential.^56^ Thereby, one indirect pathway is also that the OH˙ radical is formed (detail equated below) and carries out the decomposition of TCH. The effect of both these species on the degradation process is well established *via* scavenger experiments. The designed Scheme 1 navigates the reduction (N_2_ to NH_3_) and degradation (TCH) pathway.

Eqn (2) and (3) for the indirect hydroxyl radical generation are narrated as follows:

2 O_2_ + e^−^ → ˙O_2_^−^, ˙O_2_^−^ + H^+^ → H_2_O_2_ + O_2_

3 H_2_O_2_ + O_2_ + ˙O_2_^−^ → OH˙ + O_2_ + OH^−^

Additionally, the eqn (4) depicts the N_2_ reduction.^57^

4 N_2_ + 6H^+^ + 6e^−^ → 2NH_3_

## Conclusion

In this study, we demonstrated a successful synthetic protocol for carbonised TiO_2_ and its doped counterparts and further verified their catalytic ability by exposing these fabricated photocatalysts towards dinitrogen photoreduction and TCH degradation under a visible light source. It was observed that of our prepared catalysts, B-CT displays the best activity, *i.e.* a 0.076% conversion efficiency towards nitrogen photo-fixation and 95% TCH degradation, which is well supported by the PL, BET and EIS data. The above characterization techniques clearly indicate a better charge separation and more active site availability in B-CT, which result in its high catalytic activity. Further, more defects in the formed B-CT can be well justified by XPS and Urbach energy analyses, which also play a crucial role in its increase in activity. The synthesis route is expected to be applicable in designing other anions doped with defect-oriented metal oxide 2D systems. Additionally, this investigation may open up a new avenue for exploring various hybrids of defect framed doped TiO_2_, which would be effective towards environmental sustainability and energy-related applications.

## Conflicts of interest

There is no conflict of interest.

## Supplementary Material

NA-002-D0NA00183J-s001
