# Supplementary material for: Bandgap engineering via boron and sulphur doped carbon modified anatase TiO2: a visible light stimulated photocatalyst for photo-fixation of N2 and TCH degradation
Source: Nanoscale Adv. 2020 Mar 31;2(5):2004–17. doi: 10.1039/d0na00183j (PMC9419573; doi:10.1039/d0na00183j)
Supplement: NA-002-D0NA00183J-s001 [file NA-002-D0NA00183J-s001.pdf]

Electronic Supplementary Material (ESI) for Nanoscale Advances.
This journal is © The Royal Society of Chemistry 2020

Bandgap engineering via Boron and Sulphur doped carbon modified Anatase TiO_2: A Visible light stimulated Photocatalyst for Photo-Fixation of N_2 and TCH Degradation

Centre for Nano Science and Nano Technology, Siksha 'O' Anusandhan Deemed to be University, Bhubaneswar 751 030, Odisha, India

*Corresponding author

E-mail: paridakulamani@yahoo.com and

kulamaniparida@soa.ac.in

Telephone +91-674-2379425, Fax. +91-6 74-2581637.

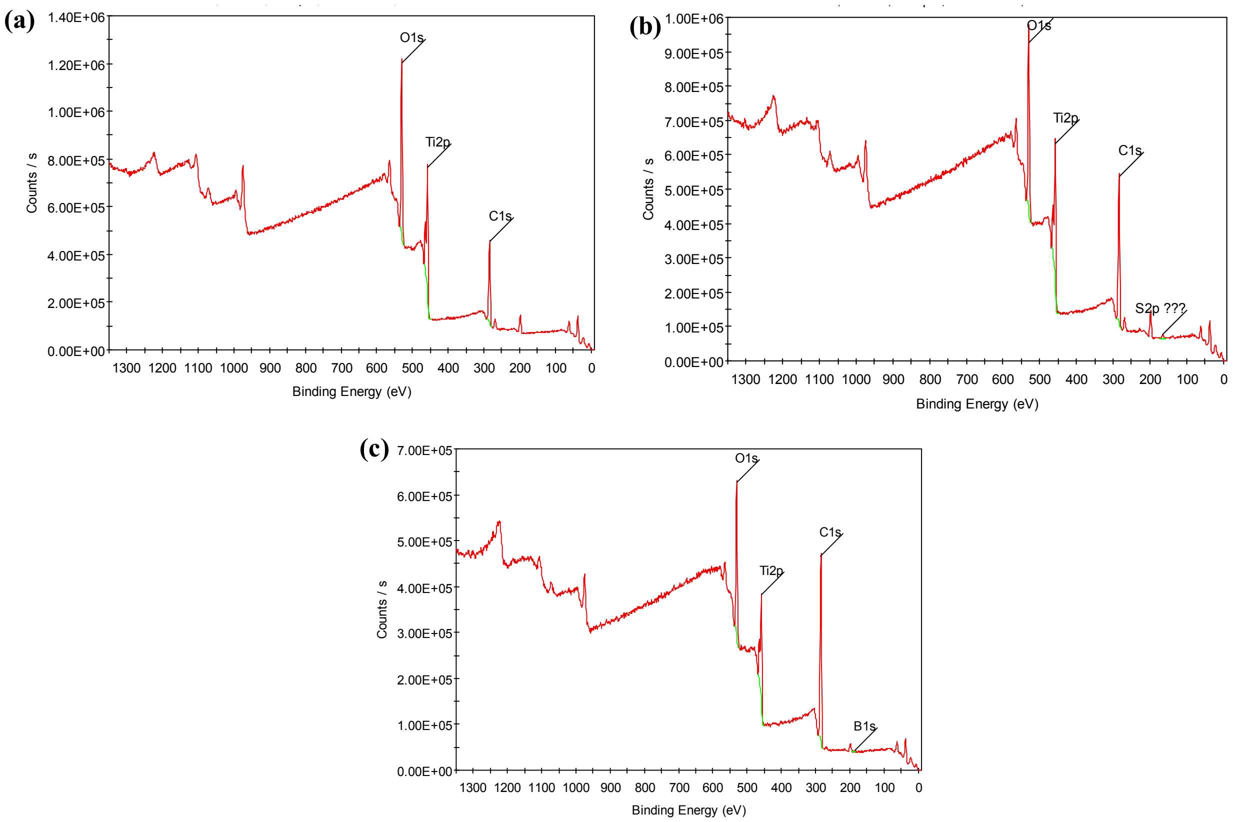

Figure. S1 XPS survey spectrum of (a) CT, (b) S-CT and (c) B-CT.

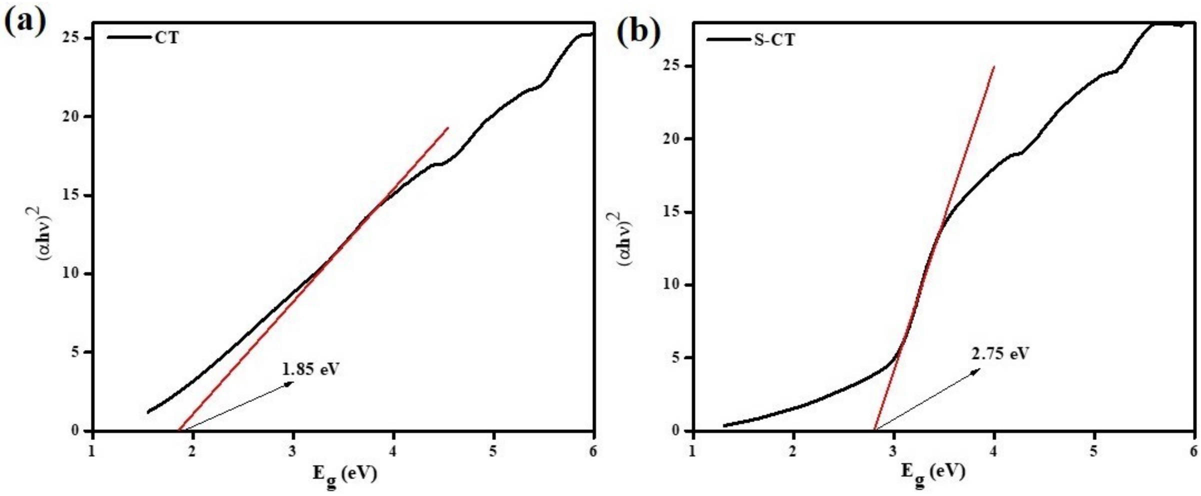

Figure. S2 Bandgap energy of (a) CT and (b) S-CT

Urbach energy calculation

Following the mathematics $\alpha = \alpha_0 \exp[E/E_u]$, where α stands for absorption coefficient, E as the energy of photon and E_u represents Urbach energy respectively. [1,2] As the absorption coefficient is equal to absorbance (A or F(R)) for solid samples, so α can be substituted by A. [2] The Urbach energy was calculated by taking the reciprocal of the slope of the graph that is plotted between lnA vs E.

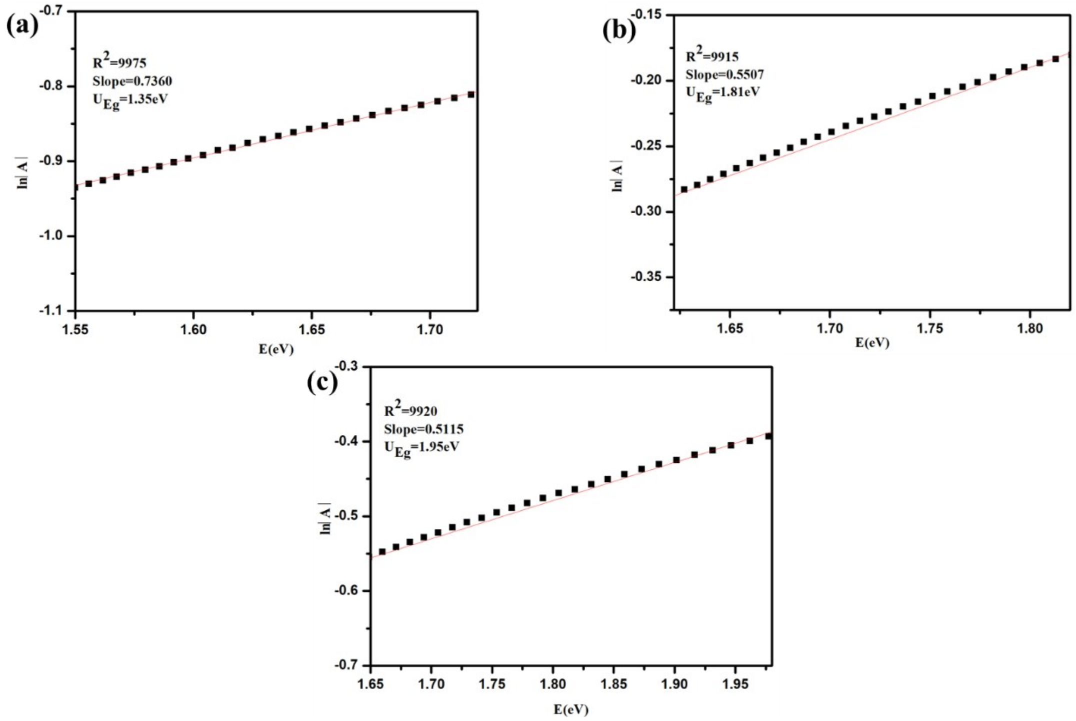

Figure. S3 Urbach energy (a) CT, (b) S-CT and (c) B-CT.

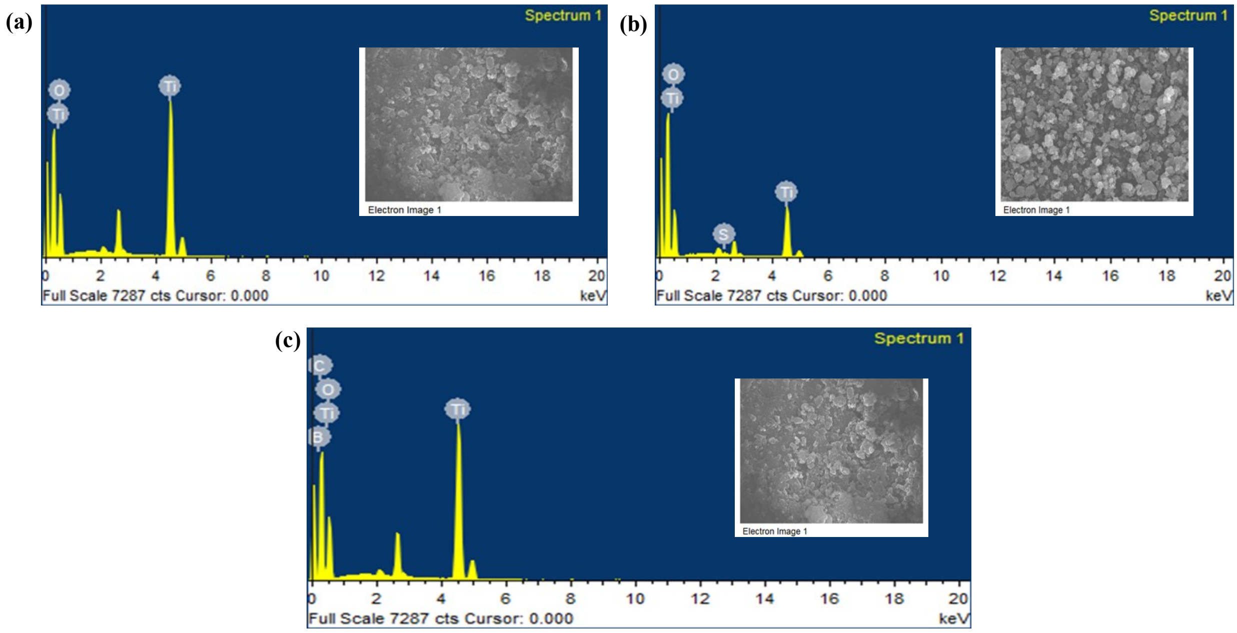

Figure. S4 EDX of (a) CT, (b) S-CT and (c) B-CT.

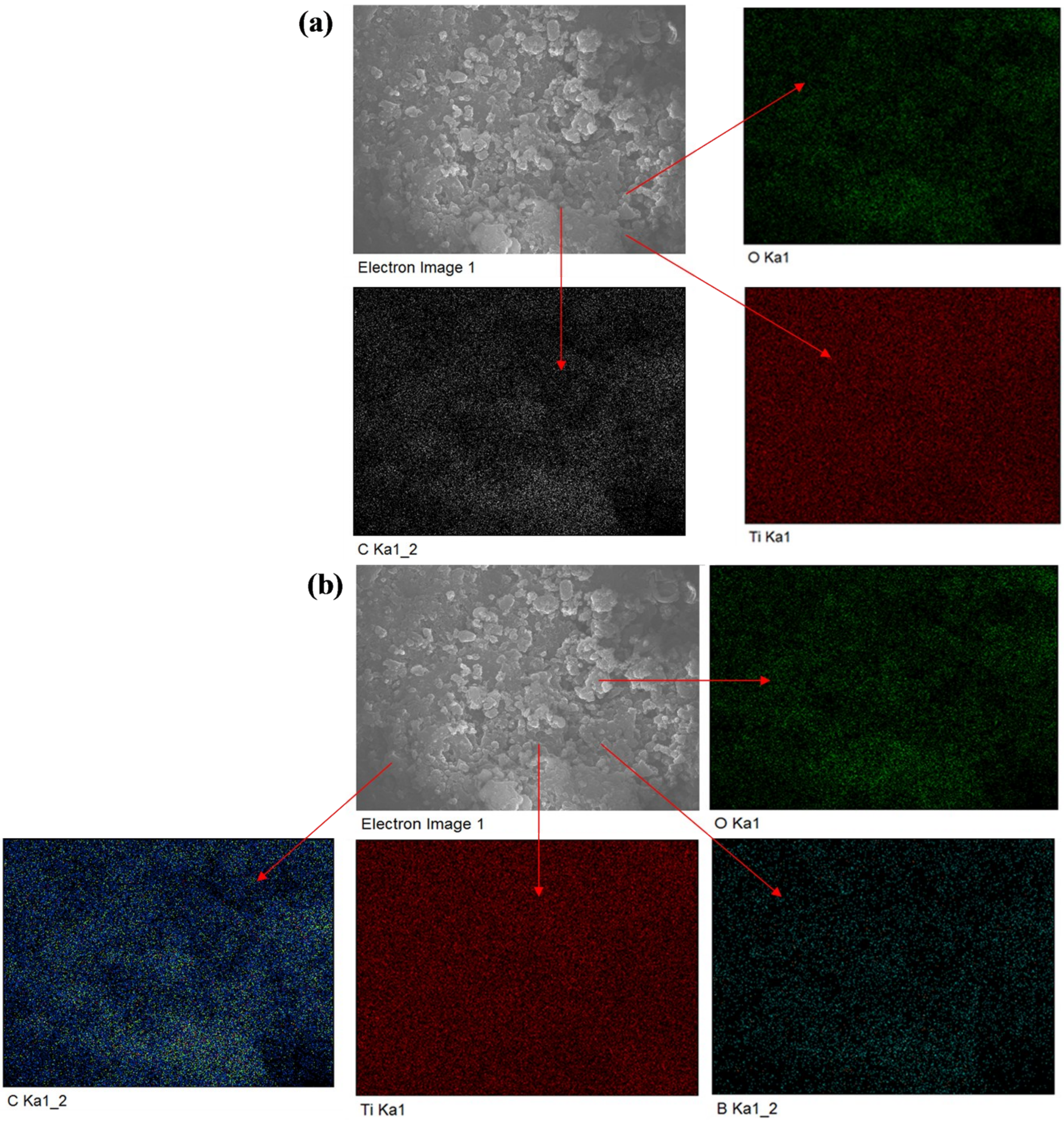

Figure. S5 Elemental colour mapping of (a) CT and (b) B-CT.

Catalytic system	Antibiotic type	Degradation%	Light source	Time period	Reference
Au/B-TiO_2/rGO	15ppm TCH	100	300W Xe lamp	60min	3
Cu-TiO2@functionalized SWCNT	30ppm sulfamethazine	100	Open sunlight	180min	4
Al-doped TiO_2	($2*10^{-4}$M)	93	(103800	120min	5

Nanoflakes	Fluoroquinolone		lux as per HTC LX102A lux meter		
Co-TNs/rGO	30ppm TCH	60	Halogen, ECO OSRAM 500 W lamp	180min	6
N-doped TiO_2	1.0×10^{-2} mol L^{-1} cefazolin	80	5 × 8W blacklight fluorescent lamps	30min	7
Co-doped TiO_2	10ppm	>90	Xe lamp	240 min of irradiation under UV-C and 300 min under visible irradiation	8
Carbon-sensitized and nitrogen-doped TiO_2	5ppm	95	LED flexible strip (SMD 5016 water research 45 (2011) 5015 - 5026 5050, 15W)	5h	9
Nitrogen-doped TiO_2/diatomite	20ppm	91	150W Xe lamp	90min	10
B-CT	10ppm	95	250W Xe-lamp	60min	Present work

Table. T1 comparison table representing different doped TiO_2 towards antibiotic degradation.

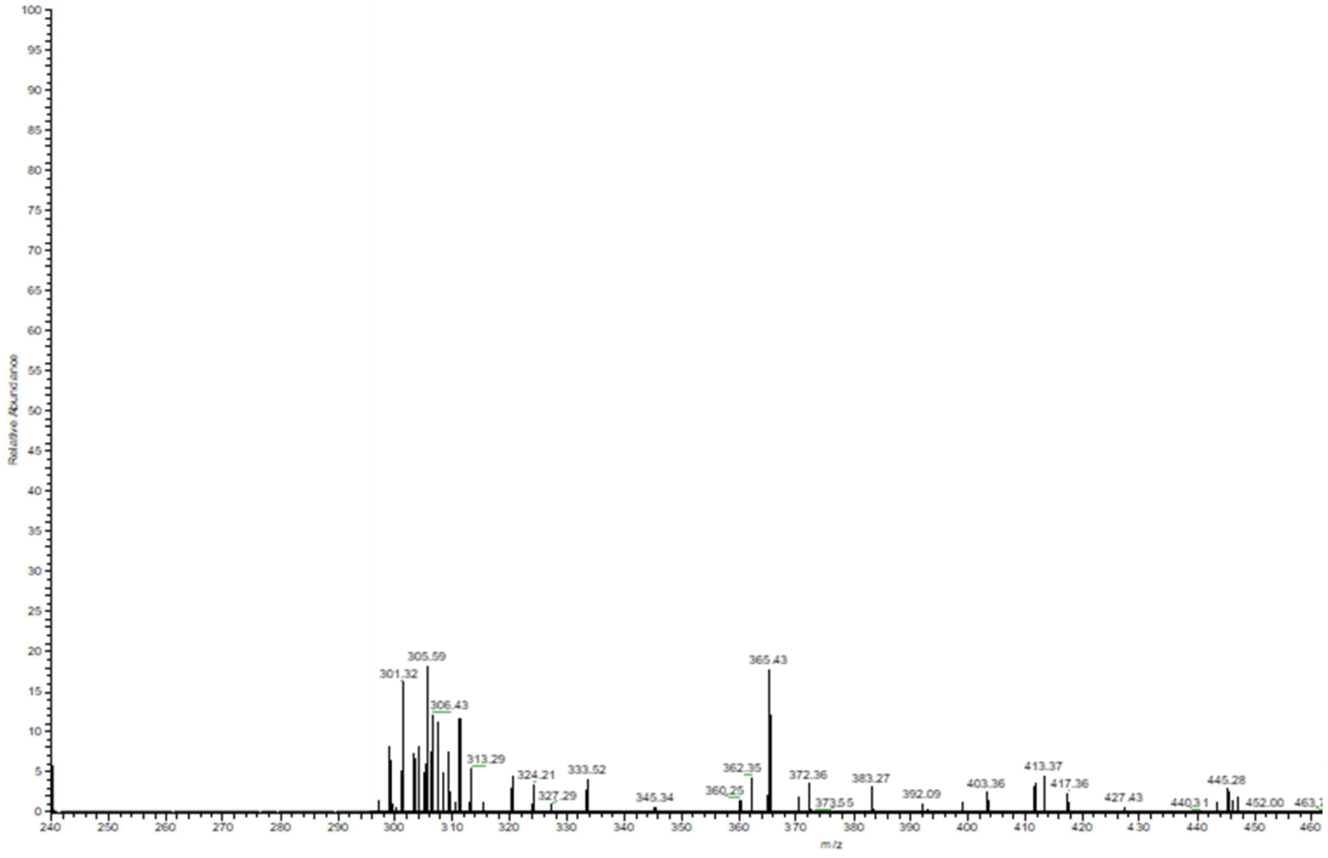

Figure. S6 LC-MS chromatogram of TCH over B-CT.

References

1. Choudhury, B.; Chetri P.; Choudhury, A., Annealing temperature and oxygen vacancy dependent variation of lattice strain, band gap and luminescence properties of CeO_2 nanoparticles. *J. Exp. Nanosci.* **2015**, *10,*103-114.
2. Deng H.; Hossenlopp, J. M., Combined X-ray diffraction and diffuse reflectance analysis of nanocrystalline mixed Sn (II) and Sn (IV) oxide powders. J. M., *J. Phys. Chem. B,* **2005**, *109,* 66-73.
3. Vinesh, V.; Shaheer A.R.M.; Neppolian, B., Reduced graphene oxide (rGO) supported electron deficient B-doped TiO_2 (Au/B-TiO_2/rGO) nanocomposite: An efficient visible light sonophotocatalyst for the degradation of Tetracycline (TC). *Ultrason. Sonochem.,* **2019**, *50*, 302-310.
4. Payan, A.; Isari A.A.; Gholizade, N.; Catalytic decomposition of sulfamethazine antibiotic and pharmaceutical wastewater using Cu-TiO_2@ functionalized SWCNT

ternary porous nanocomposite: Influential factors, mechanism, and pathway studies. *Chem. Eng. J.*, **2019***, 361*,1121-1141.

5. Kaushik, R.; Samal, P.K.; Halder, A., Degradation of Fluoroquinolone-Based Pollutants and Bacterial Inactivation by Visible-Light-Active Aluminum-Doped TiO_2 Nanoflakes. *ACS Appl. Nano Mater.*, **2019**, *2,* 7898-7909.
6. Alyani, S.J.; Pirbazari, A.E.; Khalilsaraei, F.E.; Kolur, N.A.; Gilani, N., Growing Co-doped TiO_2 nanosheets on reduced graphene oxide for efficient photocatalytic removal of tetracycline antibiotic from aqueous solution and modeling the process by artificial neural network. *J. Alloys Compd*., **2019***, 799*, 169-182.
7. Gurkan, Y.Y.; Turkten, N.; Hatipoglu, A.; Cinar, Z., Photocatalytic degradation of cefazolin over N-doped TiO_2 under UV and sunlight irradiation: Prediction of the reaction paths via conceptual DFT. *Chem. Eng. J.*, **2012**, *184*, 113-124.
8. Çağlar Yılmaz, H.; Akgeyik, E.; Bougarrani, S.; El Azzouzi M.; Erdemoğlu, S., Photocatalytic degradation of amoxicillin using Co-doped TiO_2 synthesized by reflux method and monitoring of degradation products by LC–MS/MS. *J. Dispersion Sci. Technol.,* **2020**, *41*, 414- 425.
9. Wang, P.; Zhou, T.; Wang R.; Lim, T.T., Carbon-sensitized and nitrogen-doped TiO_2 for photocatalytic degradation of sulfanilamide under visible-light irradiation. *Water Res.*, **2011***, 45*, 5015-5026.
10. Chen Y.; Liu, K., Preparation and characterization of nitrogen-doped TiO_2/diatomite integrated photocatalytic pellet for the adsorption-degradation of tetracycline hydrochloride using visible light. *Chem. Eng. J.*, **2016**, *302*, 682-696.
